# Resuscitative Endovascular Balloon Occlusion of the Aorta (REBOA): update and insights into current practices and future directions for research and implementation

**DOI:** 10.1186/s13049-020-00807-9

**Published:** 2021-01-06

**Authors:** Marianne A. Thrailkill, Kevin H. Gladin, Catherine R. Thorpe, Teryn R. Roberts, Jae H. Choi, Kevin K. Chung, Corina N. Necsoiu, Todd E. Rasmussen, Leopoldo C. Cancio, Andriy I. Batchinsky

**Affiliations:** 1 Glacier Technical Solutions, El Paso, TX USA; 2 Extracorporeal Life Support Capability Area, United States Army Institute of Surgical Research, JBSA Ft. Sam Houston, San Antonio, TX 78234 USA; 3 United States Air Force, Bethesda, MD USA; 4 Oak Ridge Institute for Science and Education, Oak Ridge, TN USA; 5 Autonomous Reanimation and Evacuation Research Program, The Geneva Foundation, San Antonio, TX USA; 6 Uniformed Services University of the Health Sciences, Bethesda, MD USA; 7 Prolonged Field Care Capability Area, United States Army Institute of Surgical Research, JBSA Ft. Sam Houston, San Antonio, TX USA; 8 United States Army Institute of Surgical Research, JBSA Ft. Sam Houston, San Antonio, TX USA

**Keywords:** REBOA, Non-compressible torso hemorrhage

## Abstract

**Background:**

In this review, we assess the state of Resuscitative Endovascular Occlusion of the Aorta (REBOA) today with respect to out-of-hospital (OOH) vs. inhospital (H) use in blunt and penetrating trauma, as well as discuss areas of promising research that may be key in further advancement of REBOA applications.

**Methods:**

To analyze the trends in REBOA use, we conducted a review of the literature and identified articles with human or animal data that fit the respective inclusion and exclusion criteria. In separate tables, we compiled data extracted from selected articles in categories including injury type, zone and duration of REBOA, setting in which REBOA was performed, sample size, age, sex and outcome. Based on these tables as well as more detailed review of some key cases of REBOA usage, we assessed the current state of REBOA as well as coagulation and histological disturbances associated with its usage. All statistical tests were 2-sided using an alpha=0.05 for significance. Analysis was done using SAS 9.5 (Cary, NC). Tests for significance was done with a t-test for continuous data and a Chi Square Test for categorical data.

**Results:**

In a total of 44 cases performed outside of a hospital in both military and civilian settings, the overall survival was found to be 88.6%, significantly higher than the 50.4% survival calculated from 1,807 cases of REBOA performed within a hospital (p<.0001). We observe from human data a propensity to use Zone I in penetrating trauma and Zone III in blunt injuries. We observe lower final metabolic markers in animal studies with shorter REBOA time and longer follow-up times.

**Conclusions:**

Further research related to human use of REBOA must be focused on earlier initiation of REBOA after injury which may depend on development of rapid vascular access devices and techniques more so than on any new improvements in REBOA. Future animal studies should provide detailed multisystem organ assessment to accurately define organ injury and metabolic burden associated with REBOA application. Overall, animal studies must involve realistic models of injury with severe clinical scenarios approximating human trauma and exsanguination, especially with long-term follow-up after injury.

## Introduction

Intravascular occlusion to control hemorrhage was first described during the Korean War by Lieutenant Colonel Hughes, who used an intra-aortic balloon to manage hemorrhage in two patients. Hughes postulated that an earlier intervention using this method could have been beneficial, and potentially life-saving, paving the way for utilization of an intravascular hemorrhage-control capability as a form of internal tourniquet and life-saving intervention during exsanguination [1]. Decades later, this initial vision for the management of difficult-to-control bleeding is generally still valid today.

Numerous studies were performed in the subsequent decades and raised concerns of complications from aortic blood flow occlusion, such as mesenteric and lower body ischemia and renal injury to name a few. Instead of balloon occlusion, resuscitative thoracotomy (RT) with a descending aortic clamp was adopted as a standard method of controlling exsanguinating hemorrhage in patients in extremis, and was shown to improve survival, despite caution regarding invasiveness not dissimilar to that of balloon occlusion [2–4]. Thus, despite the fact that RT involved invasive surgical access and was accompanied by the same complications that slowed the adoption of aortic balloon occlusion, RT was the preferred method due to greater provider familiarity. These and other aspects of balloon occlusion development were detailed in numerous reviews [5–7].

**Fig. 1:**
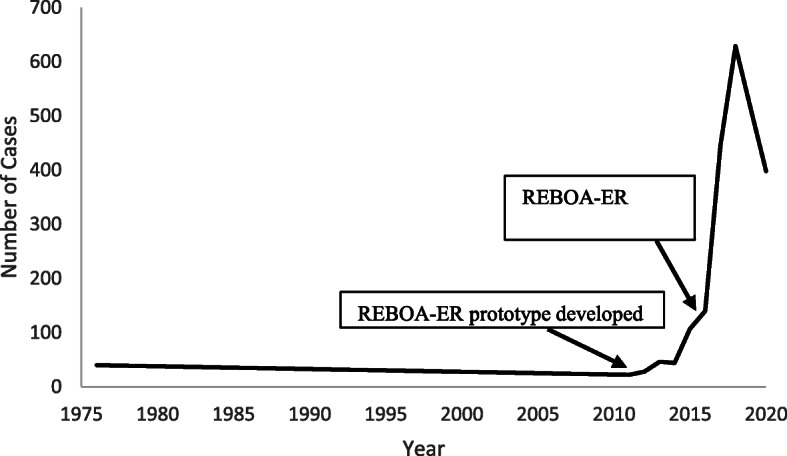
Graph depicting total number of resuscitative endovascular balloon occlusion of the aorta (REBOA) cases per year based on literature review of human and animal studies. Arrows denote the years 2011, when the REBOA prototype was developed, and 2016, in which the REBOA-ER® was cleared by the U.S. Food and Drug Administration. Data generated using original human and animal REBOA studies published in the literature with exception of databases with overlapping sources

Beginning in 2010, improvements in balloon technology and a series of encouraging pre-clinical studies led to wider implementation of “Resuscitative Endovascular Balloon Occlusion of the Aorta” (REBOA). Importantly, the appearance of purpose-designed prototypes circa 2011–2012, followed by U.S. Food and Drug Administration (FDA) clearance of the REBOA-ER® (Prytime Medical Inc., Boerne, TX) in 2016, all led to a significant inflection point (Fig. 1) in utilization of endovascular balloon technology specifically with the purpose of hemorrhage control.

Together with pioneering activities within the U.S. military, the availability of compact REBOA-specific technology ushered in a new era of translational and clinical research well summarized in recent reviews [7–11]. The purpose of this manuscript is to assess the state of REBOA today with respect to out-of-hospital (OOH) vs. in-hospital (H) use in blunt and penetrating trauma, as well as to discuss areas of promising research that may be key in further advancement of REBOA applications. We also report on common metabolic markers during REBOA use in animals and revisit certain ischemia-reperfusion, coagulation and histological disturbances associated with REBOA use.

### Current use of REBOA

Originally conceived to manage non-compressible torso hemorrhage, the indications for the use of REBOA have come to address a broad array of morbidities, due to its ability to induce occlusion of the aorta in multiple locations or “Zones”. Deployment of REBOA in the supra-diaphragmatic location (Zone I) allows for control of lower-torso/abdominal or lower extremity bleeding. Zone II deployment is problematic as it is highly dependent on accurate placement between the celiac trunk and the renal arteries (Zone II), which is difficult to achieve without contrast/visualization. Zone III involves placement below the renal arteries but proximal to the iliac bifurcation. This is arguably the least complicated of the three zones, and is intended for control of bleeding in the pelvis and extremities [12]. Consensus opinions indicate that shorter periods of occlusion which minimize ischemic injury are best for patient survival, with Zone III generally allowing for longer periods of occlusion than Zone I (60–90 min for Zone III vs. 30–60 for Zone I) [13].

Over the last decade REBOA progressed to intense investigation in multiple clinical trials (Table 1).

**Table 1:** Summary of current clinical trials as listed on clinicaltrials.gov. Year refers to the year the study was posted

Year	Trial Identifier	Title	Status
2018	NCT03534011	Resuscitative Balloon Occlusion of the Aorta in Non-traumatic Out of Hospital Cardiac Arrest (REBOA)	Currently Recruiting
2018	NCT03664557	Feasibility of REBOA in Refractory Cardiac Arrest	Completed
2018	NCT03703453	Resuscitative EndoVascular Aortic Occlusion for Maximum Perfusion	Active, Not Recruiting
2019	NCT04145271	Pre-Hospital Zone 1 Partial Resuscitative Endovascular Balloon Occlusion of the Aorta (REBOA) (PREBOA)	Not Yet Recruiting
2019	NCT03977168	A Prospective Study of Early Mechanical Stabilization and Bleeding in Disruption of the Pelvic Ring (EMS-BIND)	Recruiting by Invitation Only
2020	NCT04373122	REBOA in Out-of-hospital Cardiac Arrest	Not Yet Recruiting
2020	NCT04491903	NEURESCUE for Out-of-Hospital Cardiac Arrest	Not Yet Recruiting

The 7 clinical studies mirror the current interest in the clinical community which can be summarized as: 1) early out-of-hospital use of REBOA for blunt and penetrating trauma to include expanded use in cardiac arrest; 2) in-hospital use of REBOA and optimization of its use; 3) duration of safe REBOA use and mitigation of ischemia reperfusion injury; and 4) experimental development of partial or intermittent REBOA use.

Partial or intermittent REBOA involves balloon inflation to a degree (usually a target blood pressure or balloon volume) followed by partial or intermittent deflation. This procedure aims to temporarily restore blood flow or to provide cycles of inflation-deflation in order to buy time and avoid prolonged ischemia. However, these areas of clinical focus present challenges which are very well reviewed by Bulger et al. [10].

By looking at the decline in peak REBOA cases in 2019–2020 (Fig. 1) and the slow progress with patient enrollment in the clinical trials (Table 1), it is evident that REBOA science may be at a crossroad. To continue the momentum, patient selection and intervention timing must be addressed. Improvements are also needed in vascular access technique, teamwork, and training [11].

International registry studies by Norii et al. showed that of the 45,531 patients who met inclusion criteria, 452 patients (with a median Injury Severity Score [ISS] of 35) underwent REBOA placement. This group had a high mortality rate (76%) when compared to a much-less-injured group that did not receive REBOA (median ISS 13, *p* < 0.0001; mortality 6%) [64]. The authors acknowledged that REBOA may have been used too late and as a last-ditch effort. Two years later, the same group reported 53.3 and 38.5% survival to discharge rates in severely injured young (ISS, 41) and adolescent (ISS, 38) trauma patients managed with REBOA [50]. A Japanese Trauma Data Bank study conducted by Inoue et al. utilized propensity-score matching to compare two groups of 625 hemodynamically unstable torso trauma patients treated with or without REBOA. The study showed that the in-hospital mortality was significantly higher in REBOA subjects (61.8% vs. 45.3%). The authors attribute this difference to delays with time-to-primary surgery/definitive hemostasis, which, although shorter than in the without-REBOA group, exceeded 60 min in 79% of REBOA patients [65]. Thus, neither the Norii nor Inoue studies were favorable when REBOA was initiated in-hospital and too late after injury, delaying definitive hemorrhage control. Additionally, in both of these studies, the zones of REBOA placement were undefined.

In a case series of 6 trauma patients that received Zone I and Zone III REBOA, Brenner et al. showed a very short 18-min occlusion time, signifying a faster arrival at definitive hemostasis without hemorrhage-related mortality [44]. Interestingly, Vella et al. reported lower mortality in cases of REBOA performed in the operating room (OR) compared to cases of REBOA performed in the emergency department (ED) (36.2% vs. 68.8%, *p* < 0.001), despite requiring more time to reach surgical hemostasis (116 vs. 79 min, *p* = 0.01) and increased duration of REBOA (75 vs. 23 min, *p* < 0.001) in the operating room [66]. These studies indicate the importance of continued research on the time to REBOA initiation and REBOA duration for specific indications.

In contrast to the previously discussed studies which did not differentiate REBOA by zone when determining mortality, Perkins et al. assessed the impact of REBOA placement zone in 183 REBOA patients. The survival rate for cases with Zone I placement was 39.4% while that of cases with Zone III placement was 54%, with an overall rate of 39% regardless of REBOA placement [67]. Although these data do not have a direct comparison group, the overall mortality rate is promising in comparisons with ED thoracotomy patients with sub-diaphragmic injuries, who typically have an overall survival rate of less than 10% [68]. *We believe that distinguishing the zone-specific effects of REBOA is one of the underappreciated issues in the current literature and that these effects must be addressed in future studies.*

### Pre-hospital use of REBOA

A controversial facet of REBOA implementation is its potential for use in the pre-hospital environment. Outside of the United States, pre-hospital use of REBOA by emergency medical teams has shown promise. The first case of pre-hospital use in the civilian world was performed by the London Air Ambulance (LAA) Physician-Paramedic Team in 2016 on a 32-year-old male that had fallen 15 m and suffered a pelvic fracture. The team deployed REBOA to Zone III which improved hemodynamics, providing time for the patient to be transported to a trauma center where he underwent angioembolization of pelvic vasculature. The patient remained in hospital for 52 days, recovering fully [18]. Since then, the LAA attempted pre-hospital use of Zone III REBOA in 21 cases, largely consisting of severe trauma hemorrhage (*n* = 19). Of these cases, 62% of patients (*n* = 13) in whom REBOA was successfully deployed survived to discharge from hospital, higher than previously-reported figures for in-hospital use of REBOA [67, 69]. Additionally, in 6 patients REBOA alone was sufficient to stop hemorrhage without further intervention, possibly indicating the use of REBOA as therapeutic intervention. Finally, this study also described REBOA use in non-trauma cases (*n* = 2); it was used to prevent exsanguination and restore spontaneous circulation in patients with injuries associated with intravenous drug abuse, with a positive outcome in one patient [21].

The first pre-hospital use of Zone I REBOA was described by Lamhaut et al., in which the Service d’Aide Médicale Urgente (Paris, France) deployed Zone I REBOA in a female patient undergoing CPR with presumed intra-abdominal hemorrhage. Within 17 min of the physician’s arrival, the balloon was inflated, and within 40 min the patient arrived in an operating room--an extraordinarily short time, considering the busy traffic in Paris. The patient survived the resuscitation, though she was later transferred to palliative care due to cancer [20].

Whether used OOH or in-hospital (H), REBOA has been called a team effort regardless of the theater of application [10]. To examine the differences in OOH and H REBOA use, we conducted a review of the literature using key words including “REBOA”, “resuscitative endovascular balloon occlusion of the aorta”, and “balloon occlusion”. This initial search yielded 859 results, of which we identified 276 articles of interest that were applicable to surgical critical care in humans or animals. Of these 276, we selected all articles containing original data and separated them into two categories: human data (109) or animal data (65). The human data table (Table 2) contains data from 52 manuscripts while excluding articles with overlapping data sets, articles dealing exclusively with partial or intermittent REBOA, as well as those missing more than 2 of these variables: injury type, zone of REBOA, duration of REBOA, setting in which REBOA was performed, sample size, age, sex and outcome. The animal data inclusion criteria and results are discussed following the human data.

**Table 2:** Compiled human data from 52 papers selected from literature review

Setting		Author, Year, N	Age/Sex	Injury			Zone			Duration (min)	Survival(%)
				P	B	O	I	II	III		
OOH	Military	Manley, 2017, 4 [14]	NR/4 M	4			3		1	35	100
		Lyon, 2018, 1 [15]	25/M	1			1			34	100
		Northern, 2018, 20 [16]	18–30/NR	20			17		3	21	100
		de Schoutheete, 2018, 3 [17]	39.7/2 M, 1F	3			3			31.3	100
	Civilian	Sadek, 2016, 1 [18]	32/M		1				1	NR	100
		Rich, 2017, 1 [19]	23/F		1				1	NR	100
		Lamhaut, 2018, 1 [20]	49/F		1		1			36	100
		Lendrum, 2019, 13 [21]	32/3 M, 10F		13				13	80*	62
Hospital	ED	Okada, 2016, 1 [22]	16/M		1		1			25	100
		Teeter, 2016, 33 [23]	50/23 M, 10F	2	31		33			49†, 80‡	42^1^
		Tsurukiri, 2016, 25 [24]	69*/15 M, 10F	1	15	9	16	5	4	61	48^2^
		Conti, 2017, 1 [25]	40/M		1				1	110	100
		Maruhasi, 2017, 1 [26]	50/F		1				1	18	100
		Qazi, 2017, 1 [27]	79/F		1				1	NR	D
		Cheema, 2018, 1 [28]	Mid-50s/F		1				1	32	100
		Sato, 2018, 24 [29]	52*/17 M, 7F	1	23		24			65*	41.7
		Shoji, 2018, 10 [30]	58*/6 M, 4F	3	1	6	10			NR	60^1^
		Ozkurtul, 2019, 1 [31]	17/F		1				1	NR	D
		Shinjo, 2019, 1 [32]	75/M		1				1	NR	100
		Duchesne, 2020, 524 [33]	40*/387 M, 137F	108	405	3	359	11	151	19*	49
	OR	Ledgerwood, 1976, 40 [2]	32/34 M, 6F	38	2		NR	27†§	27.5		
		Davidson, 2016, 1 [34]	28/M	1			1			20	100
		Matsumoto, 2016, 1 [35]	37/M		1		1			25	100
		Ibrahim, 2017, 1 [36]	60/M		1		1			30 + 16	100
		Nilsson, 2017, 1 [37]	17/M		1				1	46	100
		Rosenthal, 2018, 1 [38]	19/M		1		1			NR	D
		Berg, 2019, 1 [39]	14/M			1			1	NR	100
		Khan, 2019, 1 [40]	Mid-20s/M	1			1			< 50	D
		Paradis, 2019, 1 [41]	61/M		1				1	36	100^1^
		Samlowski, 2019, 1 [42]	53/M			1	1			47	100
		Ordonez, 2020, 56 [43]	32*^, 39*°/48 M, 8F	37	19		56		(27)	40*	71.4
	Other	Brenner, 2013, 6 [44]	39.5/5 M, 1F	2	4		3		3	18	66.7
		Saito, 2015, 24 [45]	NR/NR	NR	24			21S, 35 N	29.2		
		Horer, 2016, 3 [46]	49.7/2 M, 1F	NR	2		1	> 20§	66.7		
		Uchino, 2016, 1 [47]	86/F		1				1	NR	D
		Bogert, 2017, 1 [48]	24/M		1		1			NR	100
		Bunya, 2017, 1 [49]	54/M		1		1			186	100
		Norii, 2017, 54 [50]	18/32 M, 22FF	3	51		NR	NR	42.6		
		Ogura, 2017, 34 [51]	67.5*/22 M, 12F		34		NR	NR	53		
		Brenner, 2018, 79 [52]	40/66 M, 13F	24	54		64		15	53	44
		Darrable, 2018, 16 [53]	48.7/14 M, 2F	2	11	3	16			NR	32.2
		Goodenough, 2018, 1 [54]	83/M			1	1			NR	100
		Matsumura, 2018, 109 [55]	60*/71 M, 38F	5	104		NR	63*	55^1^		
		Otsuka, 2018, 15 [56]	52.7/11 M, 4F		15		15			32.5	60
		Pieper, 2018, 32 [57]	46*/23 M, 9F		32				32	55*	41^3^
		Singh, 2019, 2 [58]	73.5/2 M			1	NR	NR	50		
		Zhang, 2019, 1 [59]	72/M			1	1			> 140	D
		Aoki, 2020, 633 [60]	54*/419 M, 214F	46	587		NR	NR	52		
		Garcia, 2020, 28 [61]	32*/22 M, 6F	28			28		(11)	41	82.1
		Matsumoto, 2020, 38 [62]	42*/27 M, 11F	3	35		29	8	1	NR	42.1
		Nagashima, 2020, 1 [63]	48/F		1		1			NR	100
Totals	OOH	44		2863.6%	1636.4%	00%	25 56.8%	00%	19 43.2%	39.6	88.6^a!^
	Hospital	1,807		30517.2%	143981.3%	251.4%	691 38.2%	241.3%	217 12%	50.1	50.4^b!^
	All	1851		33318.4%	145580.3%	251.3%	716 38.7%	241.3%	226 12.2%		

*OOH* Outside of Hospital, *ED* Emergency Department, *OR* Operating Room, *NR* Not Reported, *P* Penetrating Injury, *B* Blunt Injury, *O* Other Injury, *D* Deceased

“Survival” indicates mixed categories of outcome, including: survival of procedure, survival to next level of care, survival to discharge. Values in parentheses indicate final location for balloon placement after initial placement in a different zone

*Denotes median value (all other values are means) †Denotes value from ‘survivors’ group ‡Denotes value from ‘non-survivors’ group §Denotes value with reduced N-value ^Denotes value for ‘penetrating’ injury group °Denotes value for ‘blunt’ injury group ^1^ at 30-day follow-up ^2^ at 60-day follow-up ^3^ at 28-day follow-up

^a^Value is percentage of 44 patients in OOH group that survived

^b^Value is percentage of 1807 patients in Hospital group that survived

^!^Significant difference, *p* < .0001, significance via Chi Square Test

In a total of 44 OOH cases performed in both military and civilian setting, 70.4% were males (mean age 32 **±** 9.5 STD) with 28 cases of use in penetrating injury which were primarily treated with Zone I REBOA, and 16 cases of blunt injury applications which primarily involved Zone III placement. Among all 44 cases, 25 (56.8%) received Zone I and 19 cases (43.2%) received Zone III REBOA with a median duration of 35 min (31.3–36 IQR). Overall survival was calculated to be 88.6%.

A much larger 1807 cases of H REBOA were reported and were comprised of 71.9% males (mean age 47 **±** 19.5) of which 691 (38.2%) received Zone I REBOA, 24 (1.3%) received Zone II, 217 (12%) received Zone III REBOA. The zone of placement was not reported for 875 (48.4%) cases. Among the reported data from H patients the majority had blunt injuries (81%) and the calculated survival was 50.4% (vs. 88.6% in OOH, *p* < .0001).

Our analysis outlines some important trends. On the one hand, higher survival in the OOH setting is logical if one follows the concept of earlier intervention leading to better outcomes. Indeed such observations have been reported by Clarke et al., who showed that the probability of death in hypotensive patients that spent up to 90 min in ED before transfer to OR for laparotomy and hemorrhage control increased by 0.35% for every minute of delay in the ED [70]. Shackelford et al. demonstrated an association, regardless of performance location (prehospital or in-hospital), between time to initial blood transfusion and 24-h survival in combat casualties in Afghanistan when resuscitation was initiated in the first 15 min after MEDEVAC rescue (median time after injury 36 min. Adjusted hazard ratio, 0.17 [95% CI, 0.04 to 0.73], *P* = .02) [71]. Although the Clarke and Shackelford studies did not utilize REBOA, they confirm the long-standing importance of early administration of life-saving interventions during hemorrhage. Similarly, the Royal London Hospital indicated that nearly half their center’s fatalities during H REBOA occurred due to severe pelvic hemorrhage, resulting in exsanguination before hospital arrival [72, 73].

On the other hand, it is surprising that H REBOA led to lower cumulative survival in our analysis, as more qualified providers and abundant imaging techniques and equipment should translate into better outcomes. However, a 2016 report from the AAST AORTA registry by DuBose et al., also showed a comparatively low 28% (13 out of 46) survival in the group receiving REBOA in hospital, which was not significantly higher than patients receiving operative aortic occlusion (16%, 11 of 68) [74]. An important finding from the 2016 DuBose study is that 50% of the patients received direct cutdown for cannulation; 10% were cannulated with ultrasonographic visualization and 28% received direct percutaneous cannulation without any imaging [74]. Brenner et al. reported a similar 33% use of percutaneous access and 67% cutdowns for initiation of REBOA in 90 patients with severe exsanguination and cardiac arrest, of whom 38% survived to the operating room. However, 30-day mortalities were high, both overall (62%) and for those in cardiac arrest (90%) [52]. The similar distribution of cannulation mechanism in the DuBose and Brenner studies leads us to conjecture that a more time-consuming cannulation caused by prolonged or severe periods of hypovolemic arrest post-exsanguination decreases the likelihood of survival.. The high (30%) utilization of direct palpation/percutaneous arterial cannulation without visualization in the DuBose study, likely performed by more experienced providers or done due to presumed lack of time to get the ultrasound machine, confirms our suspicion that it is the time to cannulation that determines REBOA success more so than the venue (OOH vs. H).

Proficiency in REBOA placement may be directly related to speed and accuracy of introducer placement. Our group placed REBOA over 60 times in various animal studies with pre-cannulated femoral arteries, thus removing the vascular access problem. In one particular study, despite variability in level of training or familiarity (1 surgical resident, 1 surgeon, 1 general practitioner and 1 RN), all zone I placements were successful as verified by post-mortem CT scans [75]. Proficiency with placement was also pointed out in a 2018 update from the AAST AORTA registry in which Theodorou et al. concluded that hospitals with higher patient volumes (> 80 cases) had increased odds of successful REBOA placement vs. those with lower volumes (< 20 cases) (7.50 OR; 2.10–27.29 CI, *p* = 0.002). In summary [76] we posit that improvements in accuracy and expediency of initial vascular access will remove a significant application hurdle, improving outcomes in REBOA utilization regardless of the venue where it is applied or the level of provider training. This proposition merits prospective investigation but may be a critical determinant of continued progress in REBOA use.

Another observation from Table 2 is the propensity to use Zone I in penetrating trauma and Zone III in blunt injuries. Aside from the considerations dictated by injury location, the propensity to place REBOA into Zone I is well justified as animal work in our laboratory showed that Zone I REBOA efficiently and quickly restores central circulation and carotid flow, achieving rapid cerebrovascular resuscitation [75]. Similarly, using the ongoing AORTA study registry, Beyer et al. demonstrated that Zone I REBOA achieved significantly higher systolic blood pressure compared to Zone III (58 **±** 4 mmHg vs. 41 **±** 4 mmHg, *p* = 0.008) and concluded that Zone I REBOA was associated with hemodynamic support of maximal efficiency in hypotensive trauma patients [77].

*In summary, the last decade of REBOA use in humans led to increased case count due to technological breakthrough of dedicated REBOA catheters. Further research related to human use of REBOA must be focused on earlier initiation of REBOA after injury which may depend on development of rapid vascular access devices and techniques more so than on any new improvements in REBOA. Utilization of zone-specific REBOA in penetrating* vs. *blunt trauma, in hemorrhagic shock and exsanguination cardiac arrest must be reported and studied separately in well-defined prospective studies such as the AORTA study. Team preparedness is paramount and must involve regular training.*

### Selected insights from animal studies

Swine REBOA models have been important drivers of research and innovation and provide valuable insight into human application [9, 75, 78–81]. An advantage of translational REBOA studies conducted in animal models is that studies can be performed under controlled conditions and without risk to humans, with a high degree of success, while mimicking a real-world emergency setting. As such, it is prudent to review aspects of data generated by REBOA studies in animals.

One of the first studies involving REBOA was a 2011 study conducted by White et al. REBOA increased central aortic pressure, carotid blood flow and brain oxygenation in swine with hemorrhagic shock. The REBOA group was less acidotic with lower serum lactate and pCO_2_ levels and required less fluid (667 mL vs 2166 mL; *p* < .05) and norepinephrine (0 mcg vs 52.1 mcg; *p* < .05) [5]. The White study set the stage for almost a decade of experimentation with REBOA. Markov et al. in 2013 demonstrated similar results as White in pigs with a survivable hemorrhage model and varying REBOA duration (60 and 90 min). Compared to hemorrhaged controls, they found REBOA to be beneficial in maintaining blood pressure during shock, albeit at a cost of more metabolic derangements and organ injury [82]. The study demonstrated that prolonged REBOA is a survivable and potentially life-saving intervention in the setting of hemorrhagic shock and cardiovascular collapse in swine. In 2015, Park et al. provided a longer post-balloon deflation follow-up period when they evaluated carotid blood flow in swine subjected to 65% blood volume hemorrhage treated with 30–60 min REBOA with delayed transfusion, immediate re-infusion of the shed blood (positive controls), or no resuscitation (negative controls) [75]. With REBOA (*n* = 21), survival was 95% compared to the 71% survival rate of the positive control group (*n* = 7, *p* = 0.06) and 0% survival in negative controls. Use of REBOA resulted in faster restoration of baseline carotid blood flow (6 min vs. 20.5 min in the positive control group, *p* = 0.114). When analyzing carotid blood flow post-hemorrhage, REBOA achieved maximum flow in 3.0 min while the positive control group required a median of 9.6 min (*p* = 0.006). No REBOA-related complications were observed. These results indicate the potential for use of REBOA to achieve rapid cerebrovascular resuscitation in cases of severe hemorrhagic shock [75].

Assessment of the metabolic sequelae resulting from REBOA use at various locations reveals incomplete data. The animal studies that reported metabolic variables are not representative of the current best practice for REBOA usage in humans, often far surpassing the recommended duration of no more than 30 min in Zone I or 60 min in Zone III [10]. Accordingly, laboratory data on ischemic injury might overestimate the damage that could be caused in human usage of REBOA in urban trauma systems. On the other hand, these findings are certainly relevant to REBOA use in austere settings, such as the battlefield.

Based on evaluation of 62 manuscripts of REBOA in animals selected using the same criteria as above human studies, we constructed Table 3 to review the physiologic outcome measures reported during REBOA.

**Table 3:** Lactate, Potassium, Troponin, Creatinine, and pH reported as Mean ± SD or Mean (IQR) unless specified. Values reported as Lactate: mmol/L; Potassium mmol/L; Troponin ng/mL; Creatinine mg/dL

Author, Year	N	REBOA Use (min)	Follow Up (Hours)	Survival (%)	End Study Values of Ischemic Markers and Significance vs. Control				
					Lactate	Potassium	Troponin	Creatinine	pH
Avaro, et al. 2011 [83]	8 (25)	60	1	20	9.59 ± 1.19	6.08 ± 0.44	–	–	–
Markov, et al. 2013 [82]	6 (24)	30	54	100	1.5^m^	3.8 ± 0.4	0.04 ± 0.05	1.1 ± 0.4	–
	6 (24)	90	54	100	1.5^m^	4.0 ± 0.5	0.16 ± 0.30	1.2 ± 0.2	–
Scott, et al. 2013 [84]	16	60	48	NR	0.63 (0.21)^c^	7.66 (1.45)^c^ *	–	1.7 (0.8)^c^	7.4^c^
					0.62 (0.08)^d^	6.10 (3.27)^d^ *	–	1.5 (0.2)^d^	7.4^d^
Morrison, et al. 2014 [81]	8 (24)	60	48	87.3	9.0 ± 4.5	5.2 ± 0.8 *	–	2.6 ± 0.5 *	7.22 ± 1.45
Morrison, et al. 2014 [81]	6 (20)	30	48	100	0.5 ± 0.1	–	0.28 ± 0.24	–	7.46 ± 0.02
	8 (20)	60	48	100	0.6 ± 0.2	–	0.44 ± 0.38	–	7.40 ± 0.10
	6 (20)	90	48	100	0.6 ± 0.1	–	0.38 ± 0.49	–	7.43 ± 0.04
Tibbets, et al. 2018 [85]	12 (18)	45	4.75	100	6^h, m^	–	–	1.7 ± 0.1^h, m^	7.4^h, m^
					3^i, m^	–	–	1.8 ± 0.1^i, m^	7.5^i, m^
Williams, et al. 2018 [86]	6 (12)	45	4.75	NR	5.2 (3.7–6.8)^j^ *	–	–	5.2 (3.7–6.8)^j^	–
					3.0 (2.4–3.6)^k^ *	–	–	1.66 (1.63–1.69)^k^	–
Beyer, et al. 2019 [77]	6 (18)	45	4.75	NR	–	–	6.26 ± 5.35 *	–	–
Kauvar, et al. 2019 [9, 87]	8 (21)	60	6	37.5	19.2 ± 2.3 *	5.1 ± 0.21	58.1 ± 28.6 *	4.0 ± 0.37 *	–
Kuckelman, et al. 2019 [88]	5 (20)	60	2	20	12.8	–	–	1.8	7.22
Sadeghi, et al. 2020 [89]	6 (18)	30	3	100	5.4 (2.4–8.4)	–	NR	–	7.5
Singer, et al. 2020 [90]	20	37^a^	4^b^	NR	10^f^	5^f^	–	–	~ 7.4^f^
					7^g^	5.5^g^	–	–	~ 7.4^g^
Yamashiro, et al. 2020 [91]	6 (11)	30	3	100	3.4 ± 0.6	–	–	1.4 ± 0.1	7.43 ± 0.02
Yamashiro, et al. 2020 [91]	3 (12)	30	4	66.6	NR	–	–	1.2	NR
	5 (12)	60	4	60	5.7 *	–	–	1.7 *	7.2 *

*NR* Not reported; * denotes *p* < 0.05 – Significance measured between REBOA and control (non REBOA) group unless specified

Notes: a: Mean Value; b: Hours post-flight; c: Measured by commercial device; d: Measured by prototype device; e: Commercial device measurements higher than prototype device; f: Flight Group; g: No flight group; h: Zone I application; i: Zone III application; j: REBOA group; k: EVAC group; l: Baseline v. study endpoint; m: Exact values not reported; data only shown graphically; n: pREBOA significantly lower than REBOA; o: REBOA statistically higher than pREBOA - *p* value not reported

Although data on the metabolic consequences of REBOA are sporadic, the table gives an overview of the ranges of changes in lactate, potassium, troponin, creatinine and pH. The range of lactate numbers spans normal to clearly high values and depends on the duration of REBOA and time of follow-up, with shorter REBOA time and longer follow-up times both determining lower final metabolic markers. This is because shorter REBOA time is almost universally associated with fewer ischemia reperfusion injury complications and longer follow-up times permit for restoration of metabolic derangements after REBOA deployment. This is well evidenced in the study by Morrison et al. which reported 100% survival after 48 h of intensive care unit follow-up in 3 groups of animals with REBOA durations of 30, 60 and 90 min [81]. Normal levels of metabolic markers were reported after balloon deflation, with some transient inflammatory mediator activation (IL6) particularly in the 60- and 90-min groups as well as a tendency to require more vasopressor support (NS) and to develop acute respiratory distress syndrome (ARDS, NS) [81]. In contrast to Morrison’s experimental conditions, which uniquely focused on multi-day outcomes after REBOA, Kauvar et al. reported a relevant short study using 60 min of REBOA and 6-h follow-up after a severe combined trauma/uncontrolled hemorrhage injury [87]. Using both 60 min of complete REBOA and 15 min of 50% deflated partial REBOA, profound lactatemia, hyperkalemia and increased troponin and creatinine levels were found at end follow-up, with a combined mortality of 37.5% [87]. Other studies in Table 3 are less complete with respect to reported metabolic markers but span the range established by the Morrison and Kauvar studies. It is imperative that comprehensive metabolic marker panels are reported in animal and human studies to address the recurring questions associated with the “metabolic cost” of REBOA deployment. *In summary, the metabolic burden associated with REBOA use is not reported consistently and must be emphasized to better define the timing of REBOA utilization in the longitudinal management of injured.*

**Fig. 2:**
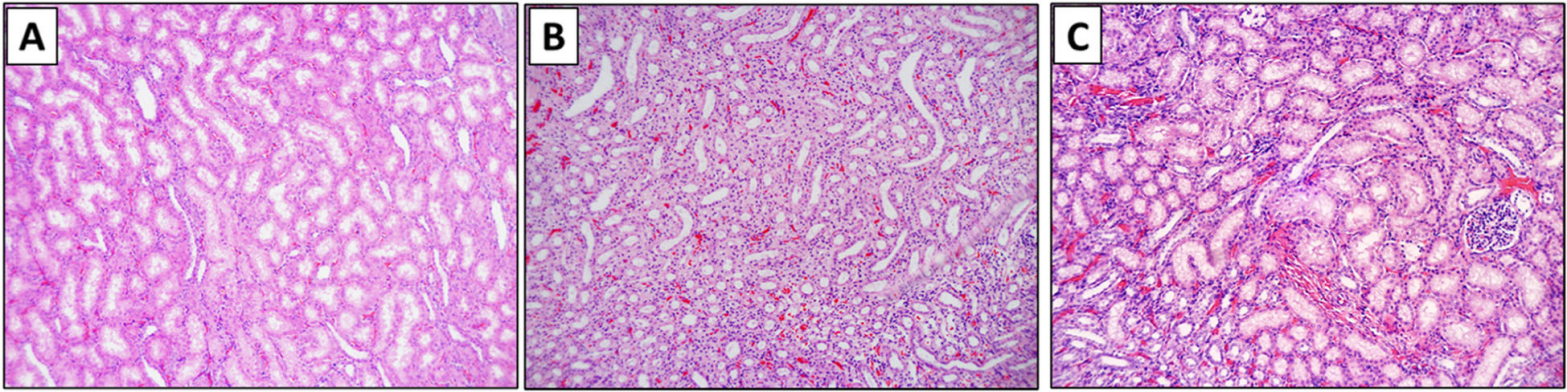
Comparison of histological appearance of kidneys in anesthetized, intubated, mechanically ventilated swine after critical care events and 6-h follow. Anesthetized mechanically ventilated swine with a mild hemorrhage (12% estimated blood volume) show only mild signs of glomerular and tubular damage (A) (no REBOA deployed). In animals that underwent 120 min of partial REBOA with target mean arterial pressure below the balloon of 45–60 mmHg, (B) more pronounced evidence of injury is present but not as severe as in an animal after 50% hemorrhage and cardiac arrest treated with 15 min REBOA and CPR (C) manifesting the most severe hemorrhage, congestion and damage to proximal and distal tubular structures and epithelium

Another example of inconsistent reporting lies with histology. Histopathological evaluation in models undergoing REBOA inflation after hemorrhage show end organ cellular damage. This is seen particularly in the 2015 study by Park et al. which indicated a significantly higher level of kidney and liver injury in groups receiving REBOA post-injury vs. groups that underwent 65% blood volume hemorrhage without intervention [75]. Similarly, increased organ and cellular damage were found in the kidneys and liver in a pilot study in swine with 50% hemorrhage and electrically-induced cardiac arrest treated with REBOA and chest compressions via the Lucas device (Physio-control Inc., Lund, Sweden) (unpublished data). Greater congestion in the kidney proximal tubule and liver as well as increased epithelial cell necrosis and hepatic degeneration was observed in the REBOA-treated group vs. untreated (Fig. 2). No significant differences between the two groups’ injury scores for the lungs, left ventricle, aorta and jejunum were found.

In swine that incurred a 25% blood volume loss, complete aortic occlusion (C-REBOA) increased organ damage compared to partial aortic occlusion (P-REBOA) in the intestinal mucosal layer. All of the animals treated with C-REBOA exhibited duodenal ischemic necrosis with mucosal loss, lamina propria congestion, and leukocyte infiltration. Additionally, 80% of the animals in the C-REBOA group were found to have acute tubular necrosis in the kidneys [92]. In a separate study conducted by Sadeghi et al., severe intestinal damage was reported in two of three groups of swine undergoing REBOA separated by duration of inflation. Both the 30-min and 60-min groups showed damage not seen in the 15-min group [89].

There are few histological reports on brain and spinal cord effects from REBOA treatment. However, Markov et al. found no significant difference in the rates of necrosis, inflammatory infiltrates, or edema observed in the brain and spinal cord of groups of swine treated with REBOA post-hemorrhagic shock [82]. Histological findings in the aorta consisted of a few fibrin strands present at the center of the catheter balloon site during Zone I placement of REBOA in swine undergoing cardiac arrest. These strands were not previously observed at the site nor in the aortas that were not in contact with the balloon, suggesting that the damage is from direct contact with the catheter. However, there were no significant alterations to the vessel wall or endothelial surface that would indicate clinical implications due to the catheter exposure/treatment [93]. Although the relative damage to other organs varies between different studies, all reports seem to conclude on presence of kidney injury of varying severity. Preliminary data from our laboratory (Fig. 2) provides a histological evaluation of the kidneys in animals receiving hemorrhage without REBOA (group A), with partial REBOA after hemorrhage (group B) or hypovolemia with subsequent electrically induced CA (group C). Some kidney injury, mild congestion and focal hemorrhage were present 6 h post-hemorrhage in the group without REBOA (A). The injury is more profound after prolonged partial REBOA (120 min) (B) and show most severe injury after profound 50% hemorrhage and CPR with REBOA inflated in place (C). It is possible that additional mechanical damage to anatomical structures below REBOA could have occurred during CPR, perhaps exacerbating the ischemic damage to the kidneys resulting from hypotension and cardiac arrest only.

*We believe that future publications should provide detailed multisystem organ assessment to accurately define organ injury after REBOA application. Overall, animal studies must involve realistic models of injury with severe clinical scenarios approximating human trauma and exsanguination. Long-term follow-up studies are desired, especially over the 72 h after injury – a current paradigm in military critical care.*

### REBOA and the coagulation system

The effects of aortic occlusion on systemic coagulation and inflammation are not well understood, as it is difficult to elucidate the effects of REBOA specifically during simultaneous trauma and hemorrhage [94]. These confounding variables also make it difficult to determine the maximum ischemic threshold during REBOA, as patients will have varying degrees of ischemia resulting from injury prior to aortic occlusion. During ischemia, impeded oxygen and nutrient delivery to tissue causes direct cellular and subcellular damage, and endothelial breakdown [95]. Platelets adhere to damaged endothelial cells and become activated, leading to fibrin cross-linking and formation of microthrombi that impede the microcirculation [96]. Fibrin and fibrin degradation products trigger leukocytes to express cytokines and stimulate ROS production [95]. Persistent activation of inflammatory pathways leads to systemic platelet activation, promoting platelet adherence to re-perfused endothelium, as well as platelet secretion of chemokines and inflammatory mediators, and exposure of surface receptors that enable platelet-leukocyte interactions [96, 97]. Simultaneous with these pro-thrombotic and inflammatory effects, suppression of anti-inflammatory and thrombolytic compounds such as activated protein C, nitric oxide and prostacyclin occurs, such that there is insufficient fibrinolytic activity relative to prothrombotic effects [98]. This cascade of events can elicit significant cellular damage, formation of intravascular thrombi, disruption of microcirculation, secondary ischemia and ultimately organ failure. *In summary, deployment of REBOA leads to non-specific coagulation disturbances associated with obstruction of flow and stasis of deoxygenated blood below the balloon.*

Partial REBOA has been investigated as a means to reduce ischemic/prothrombotic injury by allowing low-volume distal perfusion below the balloon and has been shown to reduce ischemia-reperfusion injury and regional coagulopathy when compared to complete aortic occlusion as evidenced by reduction in serum lactate and histological signs of early necrosis [99]. Similarly, intermittent REBOA reduced mortality and metabolic damage vs sustained REBOA in non-compressible torso hemorrhage in swine. Interestingly, rotational thromboelastometry showed reduced clot firmness and increased lysis in the sustained occlusion group [100]. A promising new approach was demonstrated by Necsoiu et al., who used a 50% swine hemorrhage model and compared partial REBOA using a bi-lobed catheter (consisting of a compliant and non-compliant balloons) designed for permissive hypotension to distal areas with a hypotensive target systolic blood pressure of 45 or 60 mmHg. Animals receiving this partial REBOA approach over 2 h showed restoration of cardiac output and carotid blood flow, limited ischemia-reperfusion and end-organ injury leading to significantly higher survival at 24 h vs a group with 2 h of fully inflated REBOA which showed uniform mortality [101]. Further studies are needed to understand how the duration and extent of ischemia or permissive hypotension during REBOA alters both coagulation and inflammatory outcomes enabling longer yet safe REBOA application.

In addition to partial and intermittent REBOA, therapeutic hypothermia is a potential adjuvant that has been utilized to minimize coagulation disturbances following cardiac arrest after return of spontaneous circulation. Therapeutic benefit of hypothermia during REBOA has been assessed in a large animal model of external ischemic limb cooling during 4 h of Zone III REBOA. In this study, hypothermia was localized to distal ischemic limbs while normal core body temperature was maintained. Local hypothermia reduced compartment pressures as well as serum levels of creatinine kinase and myoglobin, suggesting a reduction in ischemic damage; however, impact on coagulation was not assessed [102]. Additionally, when this model of external limb cooling was extended to 8 h of Zone III REBOA, no benefit of local hypothermia was observed and significant clot emboli occurred in the lower extremities upon balloon deflation [103]. Further study is needed to assess the impact of both local and systemic hypothermia during REBOA on ischemia-reperfusion injury and coagulation specifically. New partial REBOA approaches to achieve controlled lower body hypotension as well as the use of viscoelastic assays to assess coagulopathy are being investigated for this purpose.

*Further understanding of REBOA-associated coagulopathic complications will rely on development of deployable regional cooling and distal perfusion solutions as well as deployable tools to monitor coagulation in the field. Ideally, assessment of platelet count, prothrombin time, activated partial thromboplastin time, fibrinogen and fibrinogen degradation products using predictive models may allow for identification of coagulation abnormalities and guide resuscitative strategies.*

### Looking forward

In assessing data from both clinical use of REBOA and large animal studies, and addressing related histological and coagulative effects of REBOA, this review provides an overarching look at some relatively underreported aspects of REBOA research. Though there have been many advances in technology in both the hospital and prehospital setting, there are still challenges to the widespread use of REBOA for non-compressible hemorrhage.

One of the primary challenges affecting REBOA use is the difficulty of diagnosing the presence of hemorrhagic shock, especially in blunt trauma, NCTH but also polytrauma with traumatic brain injury component. These challenges will require better diagnostic tools and predictive assessment of bleeding degree, rate and trajectory of the patient.

Limitations of REBOA are first and foremost related to the need for technology for *rapid and accurate vessel cannulation*. Whereas many mention this point, we believe that it is of paramount importance and must be addressed as the *number one priority for future research and should be considered a rate limiting step in development of future intravascular interventions*. The vascular access challenges are of particular importance in prehospital settings where austere conditions, less experienced providers, lack of visualizing equipment, and patient status can further complicate vessel cannulation and, by extension, REBOA placement. To improve success in non-hospital settings, transportable imaging devices have been developed to confirm balloon position in lieu of fluoroscopy. Such innovations include a proof-of-concept study using radiofrequency identification to determine REBOA placement and a protocol developed for use of ultrasound with radial arterial line monitoring of blood pressure to confirm placement and to prevent over-inflation of the balloon [51, 104].

One of the most considerable limitations for cannulation is the ability to find a pulsating femoral vessel in a patient with low blood pressure and absence of clear peripheral or central pulsation. In these cases, a cut-down could be performed or new devices that help to visualize vessels and assist with cannulation should be developed. One such handheld device for automated venipuncture has been developed by a team at Rutgers University (New Brunswick, NJ) and experienced success when used to draw 5 mL of blood in humans. The machine requires a provider to identify and position the device over an appropriate vessel, at which point the device cannulates the vessel relying on images from an ultrasound probe, doing so in this study with an overall success rate of 87% (*n* = 31) and a success rate of 97% (*n* = 25) when excluding those with difficult venous access. Though further testing is needed, this represents a promising step toward remedying one of the foremost problems associated with REBOA among other emergency procedures [105].

Due to the difficulty of cannulation of a high-risk patient, there has been a lot of focus on how to properly train physicians in this procedure. The target groups for these training programs are often not limited to physicians, but extend to a wider range of providers including nursing staff and paramedics to increase the likelihood of successfully implementing a REBOA program [106]. A four-step training program was developed at St. Olav’s Hospital (Trondheim, Norway) for implementation of REBOA by prehospital personnel, specifically for cases of non-traumatic cardiac arrest occurring out of hospital. This program, with training ranging from the theoretical level to a high-fidelity simulation, included both physicians and paramedics and was evaluated through an observational study including 10 successful uses of REBOA in both indoor and outdoor pre-hospital environments. Of note, all cannulations were performed by anesthesiologists in teams of two, with an 80% success rate for cannulation on the first attempt and with two cases requiring a second attempt. Though this study represents a single center program used for one indication of REBOA, it provides a basis for future training programs of both physician and non-physician providers to successfully initiate REBOA in pre-hospital settings [107].

In addition to this, many other groups have posited different training procedures including a standardized simulation focusing on increasing procedural competence and a porcine model to practice cannulation, decreasing overall procedural time [11, 108]. In 2019, the American College of Surgeons Committee on trauma, the American College of Emergency Physicians, the National Association of Emergency Medical Services Physicians, and the National Association of Emergency Medical Technicians released a combined statement on REBOA and released guidelines for training, suggesting that a comprehensive program would include didactic and skills-based training for providers. In addition, perfused cadavers were suggested to support vascular access [10]. The authors believe that both the rapid and reliable cannulation and training of various providers can be effectively achieved with continuous utilization of live tissue models in centers where preclinical research facilities are located near hospitals.

*Overall, the above problems indirectly but significantly limit adoption of REBOA. Better visualization tools and targeted training of providers without vascular or general surgery/critical care skills is needed to enable earlier REBOA initiation to improve treatment outcomes in the long term.* As these issues are addressed, REBOA could become an increasingly achievable and vital technique in management of overt hemorrhage effectively pausing the progression of overt hemorrhage to allow for more time for life-saving interventions when time matters most.

## Conclusions

Further research related to human use of REBOA must be focused on earlier diagnosis of bleeding, accurate criteria for initiation of REBOA after injury which may depend on development of rapid vascular access devices and techniques more so than on any other new improvements in REBOA. Future animal studies should provide detailed multisystem organ assessment to accurately define organ injury and metabolic burden associated with REBOA application. New technology is needed that permits extended mitigation of ischemia reperfusion injury below the balloon increasing duration for safe use of REBOA. Overall, animal studies must involve realistic models of injury with severe clinical scenarios approximating human trauma and exsanguination, especially with long-term follow-up after injury. For the field of REBOA to continue to progress, better visualization tools with regard to cannulation and targeted training of medical providers are critical.

## Data Availability

The datasets used during the current study are available from the corresponding author on reasonable request.

## Abbreviations

AAST: American Association for the Surgery of Trauma
AORTA: Aortic Occlusion for Resuscitation in Trauma and Acute Care Surgery
ARDS: Acute Respiratory Distress Syndrome
CPR: Cardiopulmonary Resuscitation C-REBOA Complete Resuscitative Endovascular Balloon Occlusion of the Aorta
ED: Emergency Department
FDA: Food and Drug Administration
H: In-Hospital
LAA: London Air Ambulance
MEDEVAC: Medical Evacuation
NS: Not Significant
OOH: Out-of-Hospital
OR: Operating Room
P-REBOA: Partial Resuscitative Endovascular Balloon Occlusion of the Aorta
REBOA: Resuscitative Endovascular Balloon Occlusion of the Aorta
ROS: Radical Oxygen Species
RT: Resuscitative Thoracotomy
